# Clinical Significance of MLH1 Methylation and CpG Island Methylator Phenotype as Prognostic Markers in Patients with Gastric Cancer

**DOI:** 10.1371/journal.pone.0130409

**Published:** 2015-06-29

**Authors:** Kunitoshi Shigeyasu, Takeshi Nagasaka, Yoshiko Mori, Naosuke Yokomichi, Takashi Kawai, Tomokazu Fuji, Keisuke Kimura, Yuzo Umeda, Shunsuke Kagawa, Ajay Goel, Toshiyoshi Fujiwara

**Affiliations:** 1 Department of Gastroenterological Surgery, Okayama University Graduate School of Medicine, Dentistry and Pharmaceutical Sciences, Okayama, Okayama, Japan; 2 Center for Epigenetics, Cancer Prevention and Cancer Genomics, Baylor Research Institute and Charles A Sammons Cancer Center, Baylor University Medical Center, Dallas, Texas, United States of America; Howard University, UNITED STATES

## Abstract

**Background:**

To improve the outcome of patients suffering from gastric cancer, a better understanding of underlying genetic and epigenetic events in this malignancy is required. Although CpG island methylator phenotype (CIMP) and microsatellite instability (MSI) have been shown to play pivotal roles in gastric cancer pathogenesis, the clinical significance of these events on survival outcomes in patients with gastric cancer remains unknown.

**Methods:**

This study included a patient cohort with pathologically confirmed gastric cancer who had surgical resections. A cohort of 68 gastric cancers was analyzed. CIMP and MSI statuses were determined by analyzing promoter CpG island methylation status of 28 genes/loci, and genomic instability at 10 microsatellite markers, respectively. A Cox’s proportional hazards model was performed for multivariate analysis including age, stage, tumor differentiation, *KRAS* mutation status, and combined CIMP/*MLH1 *methylation status in relation to overall survival (OS).

**Results:**

By multivariate analysis, longer OS was significantly correlated with lower pathologic stage (*P* = 0.0088), better tumor differentiation (*P* = 0.0267) and CIMP-high and *MLH1 3'* methylated status (*P* = 0.0312). Stratification of CIMP status with regards to *MLH1* methylation status further enabled prediction of gastric cancer prognosis.

**Conclusions:**

CIMP and/or *MLH1* methylation status may have a potential to be prognostic biomarkers for patients with gastric cancer.

## Introduction

Gastric cancer is the second leading cause of cancer-related deaths, with about 700,000 confirmed mortalities annually worldwide, although the incidence has gradually decreased [1–3]. Gastric cancer is generally diagnosed at an advanced stage, which is the primary cause of its poor prognosis [4]. To improve the outcome of gastric cancer, identification of genetic or epigenetic events in the progression of gastric cancer is required. The most important epigenetic event in the progression of cancer is methylation of promoter CpG regions of key tumor suppressor genes. CpG islands are almost 1-kb long sequences of DNA with high guanine—cytosine content in promoter regions of the genes [5]. In contrast to normal cells, CpG islands within tumor suppressor genes in cancer cells are often hypermethylated, leading to a CpG island methylator phenotype (CIMP) [6,7]. Epigenetic silencing of tumor-related genes due to CpG island methylation has recently been reported in gastric cancer. Aberrant CpG island methylation of >100 growth-regulatory genes in gastric cancer has thus far been reported [8–24], however, the clinical significance of CIMP in gastric cancer remains unexplored and poorly understood.

In contrast, mismatch repair (MMR) deficiency in gastric cancer is also an important genetic event. Genomic instability within the number of microsatellite repeats (or microsatellites) is termed microsatellite instability (MSI). MSI is a feature caused by a defective DNA MMR system. Functional inactivation of MMR genes, such as *MLH1* or *MSH2*, by promoter methylation is responsible for the MSI-high (MSI-H) phenotype in gastric cancer. In a previous study, gastric cancer with MSI-H showed a higher frequency of antral location, intestinal subtype, lower incidence of lymph node metastasis, and improved survival, compared to microsatellite stable (MSS) or MSI-L gastric cancers [17,25–30]. However, the clinical significance of MMR deficiency in gastric cancer remains unknown.

In view of this gap in knowledge, in this study, we explored the significance of CIMP and MMR deficiency in gastric cancer and determined their contribution as prognostic markers in patients with gastric cancer.

## Materials and Methods

### Tissue specimens

This study included a cohort of patients with pathologically confirmed gastric cancer who had undergone surgical resection at Okayama University Hospital (Okayama, Japan) from 1998–2004. A total of 68 gastric cancer tissues and their matched normal gastric mucosa were analyzed. All normal gastric mucosa tissues were obtained from sites adjacent to, but at least 5 cm away from, the original tumor. All patients provided written informed consent and the study was approved by the ethical committee of the Okayama University Hospital. All patients provided written informed consent for usage of their data for future analyses. All gastric cancers and normal gastric mucosa were fresh-frozen tissue specimens, from which DNA was extracted using a QIAamp DNA Mini Kit (QIAGEN).

### MSI analysis

MSI analysis was performed by examination of 2 mononucleotide repeats (BAT25 and BAT26), 12 dinucleotide repeats (D17S250, D18S35, D18S58, D18S69, D2S123, D4S1559, D4S2381, D4S470, D5S107, D5S346, and D8S87, TP53), and one tetranucleotide repeats, MYCL, as described previously [31]. Tumors showing allelic shifts in ≥5 of 15 markers were classified as MSI-H (hereon referred to as “MSI”), and the rest were classified as microsatellite stable (MSS).

### Sodium bisulfite modification and CIMP analyses

Because frequent hypermethylation of several genes is one of the characteristic features of tumors with CIMP, we investigated the methylation status of 28 promoter CpG island-related loci (*APC*, *CACNA1G*, *CHFR*, *COX2*, *DAPK*, *DCC*, *HPP1*, *MGMT-Mp region*, *MGMT-Eh region*, *MINT1*, *MINT2*, *MINT31*, *MLH1 5'*, *MLH1 3'*, *p14*, *p16*, *RASSF1A*, *RASSF2A-region1*, *RASSF2A-region2*, *RASSF3*, *RASSF5*, *RASSF6*, *RUNX3*, *SFRP2-region1*, *SFRP2-region2*, *UNC5C*, *3OST2*, *FOXL2*), and the corresponding primer sequences are listed in S1 Table. Genomic DNA was bisulfite-modified to convert all unmethylated cytosine residues to uracils. In brief, 0.5–2.0 μg of DNA were denatured in NaOH, treated with sodium bisulfite, and purified using the Wizard DNA Clean-up System (Promega). The methylation status of each CIMP-related locus was evaluated by combined bisulfite restriction analysis (COBRA). Polymerase chain reaction (PCR) for COBRA was performed on a bisulfite-modified template DNA in a 25-μL PCR mixture containing 12.5 μL of HotStarTaq Master Mix kit (Qiagen), 0.5 μmol/L of each PCR primer, and approximately 25 ng of bisulfite-modified DNA. PCR products were digested by addition of restriction enzyme at 37°C for 12 h. The digested DNA was separated on 3% agarose gels in 1× Tris—acetate—EDTA buffer and stained with ethidium bromide. Human normal colonic DNA treated with SssI methylase (New England Biolabs) was used as a positive control for methylated alleles, and DNA from normal lymphocytes was used as a control for unmethylated alleles. Water was used as a negative PCR control to monitor PCR contamination. CIMP-high was defined as not less than 10 of the methylation of these loci.

### *KRAS* mutation analyses

Direct sequencing was performed to identify *KRAS* exon 2 (codon 12/13) mutations. PCR for *KRAS* gene was performed in a 25-μL PCR mixture containing 12.5 μL of HotStarTaq Master Mix kit with primers. The QIAquick PCR Purification kit was used to purify PCR products, and they were directly sequenced on an ABI 310 DNA sequencer [32].

### Statistical analyses

JMP software (ver 10.0, SAS Institute Inc.) was used to perform statistical analysis. Student’s t-test was used to compare continuous variables, and Fisher’s exact test was used to analyze categorical variables. Overall survival (OS) was measured from the operation date to the date of death. The Kaplan—Meier method and log-rank statistics for differences between various prognostic factors were used to estimate OS distributions. Cox proportional hazard models were used to calculate the hazard ratio (HR) with corresponding 95% confidence interval (CI). Univariate or multivariate logistic regression analysis was performed to determine the differences in HR between each group. All reported *P* values are two-sided, and *P* < 0.05 was considered to indicate statistical significance.

## Results

### Study population

In this study, we investigated 68 patients with gastric cancer. In the promoter CpGs of the *MLH1* gene, spread of CpG methylation within its 3' region was determined to be a critical for MLH1 expression [33]. A description of the patient cohort and various clinicopathological features based on sex, age, stage, tumor differentiation, MSI status, *KRAS* mutation, and *MLH1 3'* methylation is shown in Table 1. These statuses were compared between the CIMP-high and CIMP-low groups. Of these parameters, MSI status and *MLH1 3'* methylation status showed remarkable differences between the 2 groups (Table 1).

**Table 1:** Clinicopathological features of the patients according to CIMP status.

Characteristics	Total	CIMP-high (%)	CIMP-low (%)	*P*
**No. of patients**	68	30 (44.1)	38 (55.9)	
**Sex**				1.0000
Male	46	20 (43.5)	26 (56.5)	
Female	22	10 (45.4)	12 (54.6)	
**Age at surgery**				0.1975
<70	45	17 (37.8)	28 (62.2)	
≥70	23	13 (56.5)	10 (43.5)	
**Stage**				0.6311
I/II	32	13 (40.6)	19 (59.4)	
III/IV	36	17 (47.2)	19 (52.8)	
**Tumor Differentiation**				0.8073
Well/moderate	32	15 (46.9)	17 (53.1)	
Poor	36	15 (41.7)	21 (58.3)	
**MSI status**				0.0178*
MSS	58	22 (37.9)	36 (62.1)	
MSI	10	8 (80.0)	2 (20.0)	
***KRAS***				1.0000
Wild-type	65	29 (44.6)	36 (55.4)	
Mutated	3	1 (33.3)	2 (66.7)	
***MLH1* 3′ Methylation**				0.0008***
U	57	20 (35.1)	37 (64.9)	
M	11	10 (90.9)	1 (9.1)	

* P < 0.05,

*** P < 0.001

### Methylation status

**Fig 1:**
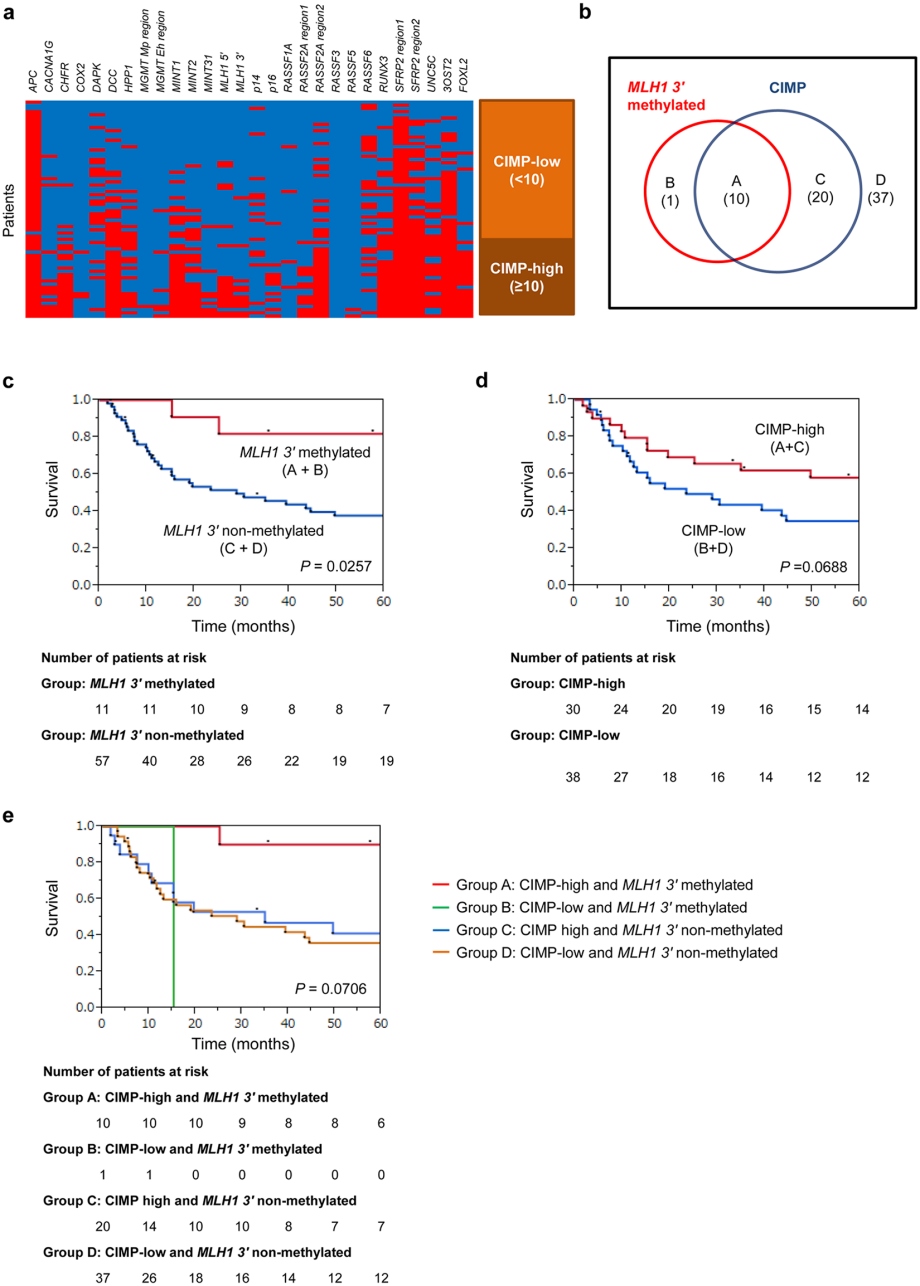
Relationship between CIMP and *MLH1* methylation. **a) Tile map showing methylation pattern**. Twenty eight loci (*APC*, *CACNA1G*, *CHFR*, *COX2*, *DAPK*, *DCC*, *HPP1*, *MGMT-Mp region*, *MGMT-Eh region*, *MINT1*, *MINT2*, *MINT31*, *MLH1 5'*, *MLH1 3'*, *p14*, *p16*, *RASSF1A*, *RASSF2A-region1*, *RASSF2A-region2*, *RASSF3*, *RASSF5*, *RASSF6*, *RUNX3*, *SFRP2-region1*, *SFRP2-region2*, *UNC5C*, *3OST2*, *FOXL2*) were analyzed to determine CIMP status. Thirty patients with not less than ten methylated loci were identified as the CIMP-high group. **b) Venn diagram showing the overlap of *MLH1* 3' methylation and CIMP status**. The overlapping relationship between *MLH1 3'* methylation and CIMP status was analyzed. 10 patients were in the combined in the CIMP-high/*MLH1 3'* methylated (A), 1 patients were in the CIMP-low/*MLH1 3'* methylated (B), 20 patients were in the CIMP high/*MLH1 3'* non-methylated (C), and 37 patients were in the CIMP-low/*MLH1 3'* non-methylated groups (D). **c) Kaplan–Meier estimate of OS in patients with *MLH1* 3' methylated or non-methylated gastric cancers**. Kaplan–Meier survival curves were generated according to *MLH1 3'* methylation status. The 5-year survival rates were analyzed for the *MLH1 3'* methylated and non-methylated groups. The survival rate was significantly higher in the *MLH1 3'* methylated group than in the non-methylated group (log-rank *P* = 0.0257). **d) Kaplan–Meier estimate of OS in patients with CIMP-high or CIMP-low gastric cancers**. Kaplan–Meier survival curves were generated according to CIMP status. The 5-year survival rate was analyzed for the CIMP-high group and CIMP-low group. The survival rate was slightly higher in the CIMP-high group than in the CIMP-low group, but the difference was not significant (log-rank *P* = 0.0688). **e) Contribution of CIMP and mismatch repair deficiency status to survival rate**. Kaplan–Meier survival curves were generated according to CIMP and *MLH1* methylation status. The patients were classified on the basis of combined CIMP status and *MLH1* methylation status into the CIMP-high/*MLH1 3'* methylated (A), CIMP-low/*MLH1 3'* methylated (B), CIMP high/*MLH1 3'* non-methylated (C), and CIMP-low/*MLH1 3'* non-methylated groups (D). Overall survival rates were higher in the combined CIMP-high/*MLH1 3'* methylated group, compared to the other groups where the differences were not statistically significant (log-rank *P* = 0.0706).

28 CpG loci including *APC*, *CACNA1G*, *CHFR*, *COX2*, *DAPK*, *DCC*, *HPP1*, *MGMT-Mp region*, *MGMT-Eh region*, *MINT1*, *MINT2*, *MINT31*, *MLH1 5'*, *MLH1 3'*, *p14*, *p16*, *RASSF1A*, *RASSF2A-region1*, *RASSF2A-region2*, *RASSF3*, *RASSF5*, *RASSF6*, *RUNX3*, *SFRP2-region1*, *SFRP2-region2*, *UNC5C*, *3OST2* and *FOXL2* were analyzed for determining the CIMP status of each gastric cancer. Methylation spectrum of these loci is shown in tile map (Fig 1a). In these loci, *CACNA1G*, *CHFR*, *DCC*, *HPP1*, *MINT1*, *MINT2*, *MINT31*, *MLH1 5'*, *MLH1 3'*, *p16*, *RASSF2A-region1*, *RASSF2A-region2*, *RUNX3*, *SFRP2-region2*, *UNC5C*, *3OST2*, and *FOXL2* were significantly methylated in the CIMP-high group (Table 2).

**Table 2:** Methylation features according to CIMP status.

	CIMP-high		CIMP-low			
Gene	Positive (%)	Negative (%)	Positive (%)	Negative (%)	Total	*P*
*APC*	28 (93.3)	2 (6.7)	36 (94.7)	2 (5.3)	68	1.0000
***CACNA1G***	**10 (33.3)**	**20 (66.7)**	**3 (7.9)**	**35 (92.1)**	**68**	**0.0122** *
***CHFR***	**22 (73.3)**	**8 (26.7)**	**1 (2.6)**	**37 (97.4)**	**68**	**<0.0001** ***
*COX2*	3 (10.0)	27 (90.0)	0 (0.0)	38 (100.0)	68	0.0810
*DAPK*	7 (23.3)	23 (76.7)	15 (39.5)	23 (60.5)	68	0.1969
***DCC***	**29 (96.7)**	**1 (3.3)**	**11 (29.0)**	**27 (71.0)**	**68**	**<0.0001** ***
***HPP1***	**17 (56.7)**	**13 (43.3)**	**4 (10.5)**	**34 (89.5)**	**68**	**<0.0001** ***
*MGMT-Mp region*	1 (3.3)	29 (96.7)	0 (0.0)	38 (100.0)	68	0.4412
*MGMT-Eh region*	5 (16.7)	25 (83.3)	1 (2.6)	37 (97.4)	68	0.0801
***MINT1***	**25 (83.3)**	**5 (16.7)**	**7 (18.4)**	**31 (81.6)**	**68**	**<0.0001** ***
***MINT2***	**21 (70.0)**	**9 (30.0)**	**4 (10.5)**	**34 (89.5)**	**68**	**<0.0001** ***
***MINT31***	**8 (26.7)**	**22 (73.3)**	**0 (0.0)**	**38 (100.0)**	**68**	**0.0008** ***
***MLH1 5*′**	**14 (46.7)**	**16 (53.3)**	**4 (10.5)**	**34 (89.5)**	**68**	**0.0018** **
***MLH1 3*′**	**10 (33.3)**	**20 (66.7)**	**1 (2.6)**	**37 (97.4)**	**68**	**0.0008** ***
*p14*	6 (20.0)	24 (80.0)	8 (21.1)	30 (78.9)	68	1.0000
***p16***	**10 (33.3)**	**20 (66.7)**	**0 (0.0)**	**38 (100.0)**	**68**	**0.0001** ***
*RASSF1A*	2 (6.67)	28 (93.3)	2 (5.3)	36 (94.7)	68	1.0000
***RASSF2A-region1***	**12 (40.0)**	**18 (60.0)**	**0 (0.0)**	**38 (100.0)**	**68**	**<0.0001** ***
***RASSF2A-region2***	**26 (86.7)**	**4 (13.3)**	**11 (29.0)**	**27 (71.0)**	**68**	**<0.0001** ***
*RASSF3*	0 (0.0)	30 (100.0)	0 (0.0)	38 (100.0)	68	1.0000
*RASSF5*	2 (6.7)	28 (93.3)	0 (0.0)	38 (100.0)	68	0.191
*RASSF6*	10 (33.3)	20 (66.7)	15 (39.5)	23 (60.5)	68	0.6234
***RUNX3***	**24 (80.0)**	**6 (20.0)**	**8 (21.1)**	**30 (78.9)**	**68**	**<0.0001** ***
*SFRP2-region1*	30 (100.0)	0 (0.0)	33 (86.8)	5 (13.2)	68	0.0618
***SFRP2-region2***	**30 (100.0)**	**0 (0.0)**	**19 (50.0)**	**19 (50.0)**	**68**	**<0.0001** ***
***UNC5C***	**21 (70.0)**	**9 (30.0)**	**8 (21.1)**	**30 (78.9)**	**68**	**<0.0001** ***
***3OST2***	**30 (100.0)**	**0 (0.0)**	**22 (57.9)**	**16 (42.1)**	**68**	**<0.0001** ***
***FOXL2***	**22 (73.3)**	**8 (26.7)**	**2 (5.3)**	**36 (94.7)**	**68**	**<0.0001** ***

* P < 0.05,

** P < 0.01,

*** P < 0.001

### Survival outcomes in patients based upon *MLH1 3'* methylation and CIMP status in gastric cancers

The overlapping relationship between CIMP and *MLH1 3'* methylation status was analyzed. 10 patients were in the CIMP-high/*MLH1 3'* methylated, 1 patient was in the CIMP-low/*MLH1 3'* methylated, 20 patients were in the CIMP high/*MLH1 3'* non-methylated and 37 patients were in the CIMP-low/*MLH1 3'* non-methylated groups (Fig 1b). Kaplan–Meier survival curves were generated according to the *MLH1 3'* methylation status. The 5-year OS rates were determined for the *MLH1 3'* methylated and non-methylated groups. The 5-year OS rates were significantly higher in the *MLH1 3'* methylated group compared to the non-methylated group (log-rank *P* = 0.0257; Fig 1c).

Likewise, the 5-year OS rates were analyzed for the CIMP-high and CIMP-low groups, and the rate was slightly higher in the CIMP-high group than in the CIMP-low group but the difference was not significant (log-rank *P* = 0.0688; Fig 1d).

### Contribution of mismatch repair deficiency and CIMP status to survival rate

The 5-year survival rates were analyzed and compared among these groups; CIMP-high/*MLH1 3'* methylated, CIMP-low/*MLH1 3'* methylated, CIMP high/*MLH1 3'* non-methylated and CIMP-low/*MLH1 3'* non-methylated groups. We noted that the overall survival rates were higher in the combined CIMP-high/*MLH1 3'* methylated group, compared to the other groups where the differences were not statistically significant (log-rank *P* = 0.0706; Fig 1e).

### Relationship between *MLH1* methylation and MSI

**Fig 2:**
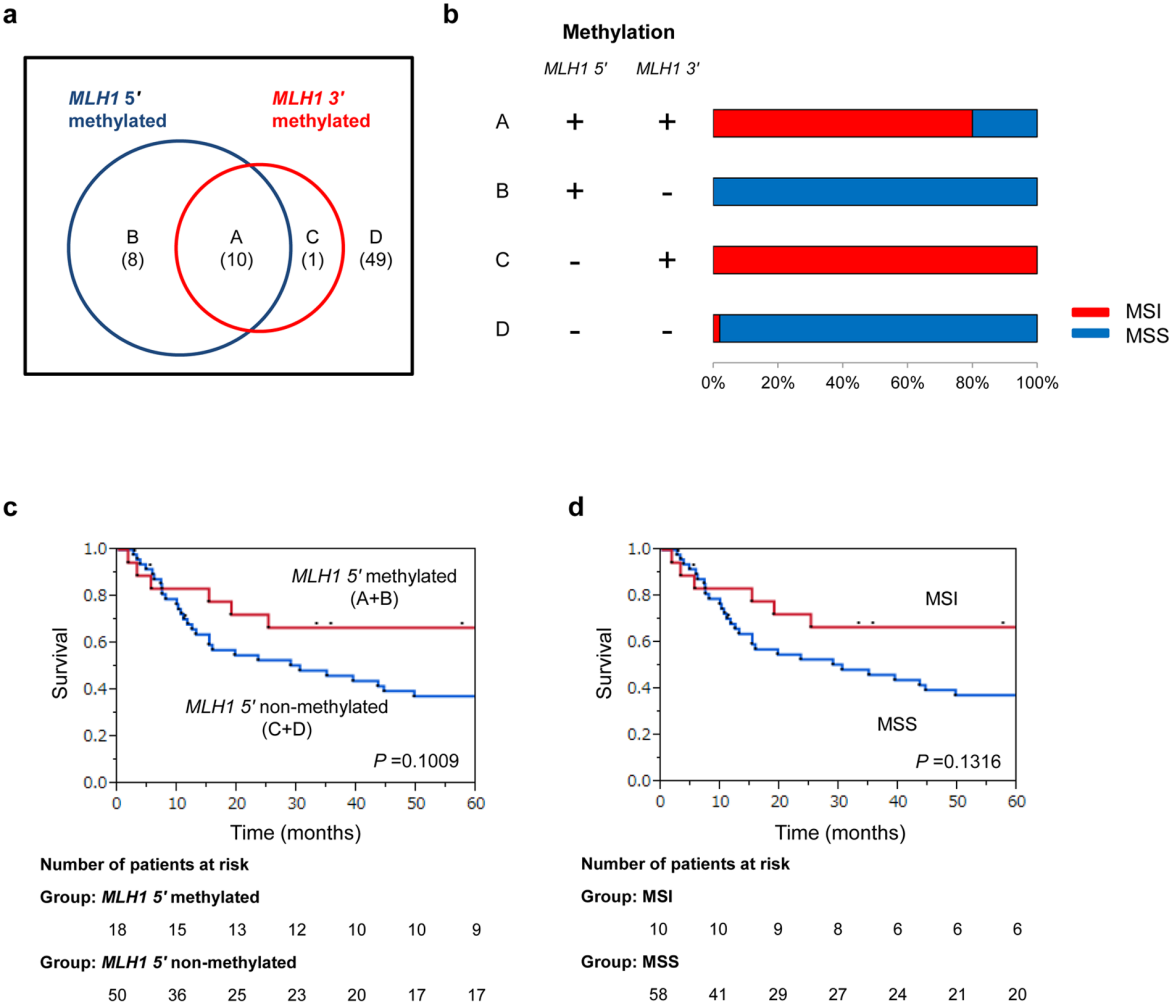
The relationship between *MLH1* methylation and MSI. **a) Venn diagram showing the overlap of MLH1 5' methylation and 3' methylation status**. The overlapping relationship between *MLH1 5'*—and *3'* methylation status was analyzed. 10 patients were in the *MLH1 5'* methylated/*3'* methylated (A), 8 patients were in the *MLH1 5'* methylated/*3'* non-methylated (B), 1 patient was in the *MLH1 5'* non-methylated/*3'* methylated (C), and 49 patients were in the *5'* non-methylated/*3'* non-methylated groups (D). **b) The relationship between *MLH1* methylation and MSI**. In the *MLH1 3'* methylated group, >80% cases showed MSI. In contrast, in the *MLH1 3'* non-methylated group, almost all cases showed MSS. **c) Kaplan–Meier estimate of OS in patients with *MLH1* 5' methylated or non-methylated gastric cancer**. Kaplan–Meier survival curves were generated according to *MLH1 5'* methylation status. The 5-year survival rate was analyzed for the *MLH1 5'* group and non-methylated groups. The survival rate was slightly higher in the *MLH1 5'* methylated group than in the non-methylated group but the difference was not significant (log-rank *P* = 0.1009). **d) Kaplan–Meier estimate of OS in patients with MSI or MSS gastric cancer**. Kaplan–Meier survival curves were generated according to MSI status. The 5-year survival rate was analyzed for the MSI group and MSS group. The survival rate was slightly higher in the MSI group than in the non-methylated group but the difference was not significant (log-rank *P* = 0.1316).

The overlapping relationship between *MLH1 5'* methylation and *3'* methylation status was analyzed. 10 patients were classified as *MLH1 5'* methylated/*3'* methylated, 8 patients as *MLH1 5'* methylated/*3'* non-methylated, 1 patient as *MLH1 5'* non-methylated/*3'* methylated, and 49 patients as *5'* non-methylated/*3'* non-methylated (Fig 2a). In the *MLH1 3'* methylated group, more than 80% patients showed MSI. In contrast, in the *MLH1 3'* non-methylated group, almost all cases showed MSS (Fig 2b). Kaplan–Meier survival curves were generated according to *MLH1 5'* methylation status. The 5-year survival rates were analyzed for the *MLH1 5'* methylated and non-methylated groups, and the rate was slightly higher in the *MLH1 5'* methylated group than in the non-methylated group but the differences were not significant (log-rank *P* = 0.1009; Fig 2c). Kaplan–Meier survival curves were generated according to MSI status. The 5-year survival rates were analyzed for the MSI and MSS groups, and the rate was slightly higher in the MSI group than in the MSS group but the differences were not significant (log-rank *P* = 0.1316; Fig 2d).

### Multivariate analysis for survival outcome predictors

A Cox proportional hazards model, including age, stage, differentiation, *KRAS* mutation status, and CIMP/*MLH1 3'* methylation status in relation to OS was used to perform multivariate analysis (Table 3). Only stage (*P* = 0.0088), differentiation (*P* = 0.0267), and CIMP/*MLH1* methylation status (*P* = 0.0312) were statistically significant predictors of OS. Hazard ratio was significantly lower in the CIMP-high/*MLH1 3'* methylated group.

**Table 3:** Multivariate analysis of outcome predictors.

		Univariate		Multivariate	
Characteristic	Total	HR (95% CI)	*P*	HR (95% CI)	*P*
**Age**			0.4476		0.4437
<70	45	1.00 (Referent)		1.00 (Referent)	
≥70	23	1.30 (0.65–2.48)		1.37 (0.60–3.03)	
**Stage**			**0.0003** ***		**0.0088** **
Stage I and II	32	1.00 (Referent)		1.00 (Referent)	
Stage III and IV	36	3.50 (1.75–7.49)		2.94 (1.31–7.09)	
**Differentiation**			**0.0032** **		**0.0267** *
Well/Moderate	32	1.00 (Referent)		1.00 (Referent)	
Poorly	36	2.76 (1.39–5.83)		2.22 (1.09–4.80)	
***KRAS* Mutation**			0.1690		0.5984
Absent	65	1.00 (Referent)		1.00 (Referent)	
Present	3	0.31 (0.02–1.47)		0.59 (0.03–3.29)	
**CIMP/*MLH1* Methylation**			**0.0311** *		**0.0312** *
CIMP−/*MLH1*-U	37	1.00 (Referent)		1.00 (Referent)	
CIMP+/*MLH1*-U	20	0.85 (0.40–1.70)	0.6535	0.67 (0.29–1.47)	0.3286
CIMP+/*MLH1*-M	10	0.19 (0.03–0.63)	**0.0042** **	0.18 (0.03–0.64)	**0.0048** **
CIMP−/*MLH1*-M	1	1.89 (0.10–9.24)	0.5722	2.11 (0.10–15.61)	0.5497

* P < 0.05,

** P < 0.01,

*** P < 0.001

## Discussion

DNA methylation in cancer cells has become a topic of intense investigation. Inactivation of tumor suppressor genes by CpG methylation of promoter regions accelerates carcinogenesis because of aberrant cell cycle regulation and proliferation [15]. Some candidate aberrantly methylated genes in gastric cancer have been reported. *RASSF1A*, *p14ARF*, and *MGMT* [34–38]; *CHRNA3*, *DOK1*, and *GNMT* [39]; *p16*, *hMLH1*, *MINT1*, *MINT2*, *MINT12*, *MINT25*, and *MINT31* [40]; *APC*, *CDH1*, *MHL1*, *CDKN2A*, *CDKN2B*, and *RUNX3* [17]; *CDH1* [41]; *DKK3* [42]; *PTEN* [43]; *MGMT*[44]; *TFPI2* [22]; *CACNA2D3* [45]; *PCDH10* [46]; *SOX2* [47]; *MAL* [48]; and *COX2* [49] were previously reported to be hypermethylated in gastric cancer. Methylation of *p16* promoter CpG islands is a marker for malignant potential of dysplasia in the stomach [50]. Methylation of *MGMT* is associated with advanced stage and poor prognosis [44]. Aberrant DNA methylation in these genes may promote development of gastric cancer. However, precise gene targets of hypermethylation for carcinogenesis remain unknown [51,52].

Concurrent CpG methylation in multiple genes has been defined as CIMP in colorectal cancer (CRC) and gastric cancer [15,40,53–57], and has been shown to correlate with hypermethylation of tumor suppressor genes. However, the evidence for CIMP in gastric cancer is not as convincing as is the case for CRC [58,59]. In gastric cancer, CIMP-high has been described in 41% [40] and 31% [54] tumors. Patients with CIMP-high gastric cancer have significantly shorter survival than those with CIMP-low gastric cancer [54,60]. Another report showed that CIMP was associated with better survival in gastric cancer [54]. The recent meta-analysis has focused on the strong relation of CIMP with H. pylori, EBV, and MSI, but CIMP could not show a prognostic potential for gastric cancer [61].

In contrast, instability at the microsatellites repeats within various growth-regulatory genes is defined as MSI. A standard panel, such as the NCI panel, is recommended, including mononucleotide (BAT26 and BAT25) and dinucleotide (D2S123, D5S346, and D17S250) repeats [62]. Three levels of MSI can be identified: high-level MSI (MSI-H), low-level MSI (MSI-L), and MSS. The MSI-H phenotype in gastric cancer was reported to account for 5%–50% MSI positive neoplasms [17]. MSI is a feature caused by a defective DNA MMR system. Functional inactivation of MMR genes, such as *MLH1* or *MSH2*, by mutational inactivation and promoter methylation is responsible for the MSI-H phenotype in gastric cancer. In particular, similar to CRC, methylation of *MLH1* is associated with the MSI-H phenotype [15,40,63,64] because *MLH1* methylation precedes the loss of protein expression. Leite M et al. reported that *MLH1* promoter hypermethylation was observed in 78.7% (70/89) of the analyzed MSI cases [65]. Methylation of the 3' region of the *MLH1* promoter, which is close to its transcriptional start site (TSS), is required for gene silencing. The 5' end of the promoter is also prone to methylation, but this is not functionally important unless the methylation extends to the critical 3' region [66,67]. MSI status is responsible for the mutation of genes regulating cell-cycle and apoptotic signaling, including *TGFβRII*, *IGFIIR*, *TCF4*, *RIZ*, *BAX*, *CASPASE5*, *FAS*, *BCL10*, and *APAF1* [17,25] and genes maintaining genomic integrity, including *MSH6*, *MSH3*, *MED1*, *RAD50*, *BLM*, *ATR*, and *MRE11* [17,68]. Gastric cancers with MSI-H show a higher frequency of antral location, intestinal subtype, lower incidence of lymph node metastasis, and improved survival relative to those of gastric cancer with MSS or MSI-L [17,25–30].

As described above, CIMP and MMR-deficiency status are key features of gastric cancer and may reflect survival differences in patients suffering from this malignancy. Significant correlation between CIMP and MSI has been reported in GC [61]. However, data regarding the synergistic effects of these parameters are scarce, and multivariate analysis of these genetic and epigenetic parameters is required. In our study, *CACNA1G*, *CHFR*, *DCC*, *HPP1*, *MINT1*, *MINT2*, *MINT31*, *MLH1 5'*, *MLH1 3'*, *p16*, *RASSF2A-region1*, *RASSF2A-region2*, *RUNX3*, *SFRP2-region2*, *UNC5C*, *3OST2*, and *FOXL2* were significantly methylated in the CIMP-high group. In particular, we reported promoter methylation of *FOXL2* in gastric cancer. *FOXL2* is a gene encoding a forkhead transcription factor and is essential for ovarian function [69]. *FOXL2* regulates the cell cycle by inducing G1 arrest and protects cells from oxidative damage by promoting oxidized DNA repair and by increasing the amount of the anti-oxidant agent glutathione [69]. *FOXL2* suppresses proliferation, invasion and promotes apoptosis of cervical cancer cells [70]. The promoter methylation of *FOXL2* may have a significant role in tumorigenesis in gastric cancer. In contrast, *MLH1 3'* methylation was required for MMR deficiency and showed MSI. The *MLH1 3'* methylation group had a tendency toward a good prognosis in the Kaplan–Meier survival estimate.

However, CIMP and MMR deficiency are dependent on each other. MSI-associated sporadic CRCs arise through a process that involves CIMP [66,71]; therefore, integrated statistical analysis of CIMP and MMR deficiency should be performed. For example, in duodenal adenocarcinomas, CIMP/*MLH1* methylation status showed a significant prognostic value in both OS and time-to-recurrence (TTR) in multivariate analysis [72]. Patients with CIMP-high/*MLH1*-unmethylated tumors had the worst OS and TTR [72]. In our multivariate analysis of patients with gastric cancer, only the CIMP-high/*MLH1 3'* methylated group had a good prognosis. The reason for good prognosis in the CIMP-high/*MLH1 3'* methylated group remains unknown. This phenomenon may be because of synergistic inactivation of vital genes due to mutation and promoter methylation. To further confirm our findings, we performed an independent validation of our results from in patient data submitted to The Cancer Genome Atlas database (TCGA). [73–75] CIMP-high/*MLH1* hyper-methylated group showed more frequent lymph node metastasis (p = 0.0009), advanced disease stage (p = 0.0078; S1 File), and slightly better Disease free survival (S2 File). On the other hand, CIMP/MSI status can’t show the significant value as prognostic marker (S2 Table). This result shows that *MLH1* has the most important role among MMR genes in the carcinogenesis of GC. Further investigation is required to elucidate the relationship between CIMP status and MMR deficiency. This approach will lead to a new strategy for the treatment of gastric cancer.

In conclusion, our data suggests that stratification of patients with CIMP based on *MLH1* methylation status may enable prediction of gastric cancer prognosis. The CIMP-high/*MLH1 3'* methylated group had good prognosis, but other groups may require intensive treatment for improvement of survival, which needs to be validated in future studies.

## Supporting Information

S1 File Relationship between CIMP and *MLH1* methylation in Gastric cancer patients in TCGA database. We investigated the methylation status of 17 promoter CpG island-related loci (*APC*, *CACNA1G*, *CHFR*, *DAPK*, *DCC*, *MGMT*, *MINT*, *MLH1*, *p16*, *RASSF1*, *RASSF2*, *RASSF3*, *RASSF5*, *RASSF6*, *RUNX3*, *SFRP2*, *and UNC5C*) in TCGA database (TCGA provisional). Upper 25% of each locus was determined as hyper-methylated. CIMP-high was defined as not less than 5 of the hyper-methylation of these loci (**Figure A**). The overlapping relationship between CIMP and *MLH1* methylation status was analyzed. 55 patients were in the CIMP-high/*MLH1* methylated, 29 patients were in the CIMP-low/*MLH1* methylated, 77 patients were in the CIMP high/*MLH1* non-methylated and 177 patients were in the CIMP-low/*MLH1* non-methylated groups (**Figure B**). Correlation between tumor depth, lymph node metastasis, distant metastasis, Stage and CIMP/*MLH1* methylation status were analyzed using Fisher’s exact test. Positive lymph node metastasis (p = 0.0009) and higher Stage (p = 0.0078) were positively correlated with CIMP-high/*MLH1* methylated group (**Figure C**).
(DOCX)

S2 File Disease free survival in Gastric cancer patients in TCGA database. Kaplan—Meier survival curves were generated according to the *MLH1* methylation status. The disease free survival rates were determined for the *MLH1* methylated and non-methylated groups. Disease free survival rates were slightly higher in the *MLH1* methylated group compared to the non-methylated group but the difference was not significant (log-rank *P* = 0.1173) (**Figure A**). Disease free survival rates were analyzed for the CIMP-high and CIMP-low groups, and the rate was slightly higher in the CIMP-high group than in the CIMP-low group but the difference was not significant (log-rank *P* = 0.1847) (**Figure B**). Disease free survival rates were analyzed and compared between CIMP-high/*MLH1* methylated and other groups. We noted that the disease free survival rates were slightly higher in the combined CIMP-high/*MLH1* methylated group, compared to the other groups where the differences were not statistically significant (log-rank P = 0.1073) (**Figure C**).
(DOCX)

S1 Table Primer sequences. (DOCX)

S2 Table Multivariate analysis of outcome predictors based on CIMP/MSI status. (DOCX)
