# Supplementary material for: Clinical Significance of MLH1 Methylation and CpG Island Methylator Phenotype as Prognostic Markers in Patients with Gastric Cancer
Source: PLoS One. 2015 Jun 29;10(6):e0130409. doi: 10.1371/journal.pone.0130409 (PMC4488282; doi:10.1371/journal.pone.0130409)

**S1 File. Relationship between CIMP and *MLH1* methylation in Gastric cancer patients in TCGA database**


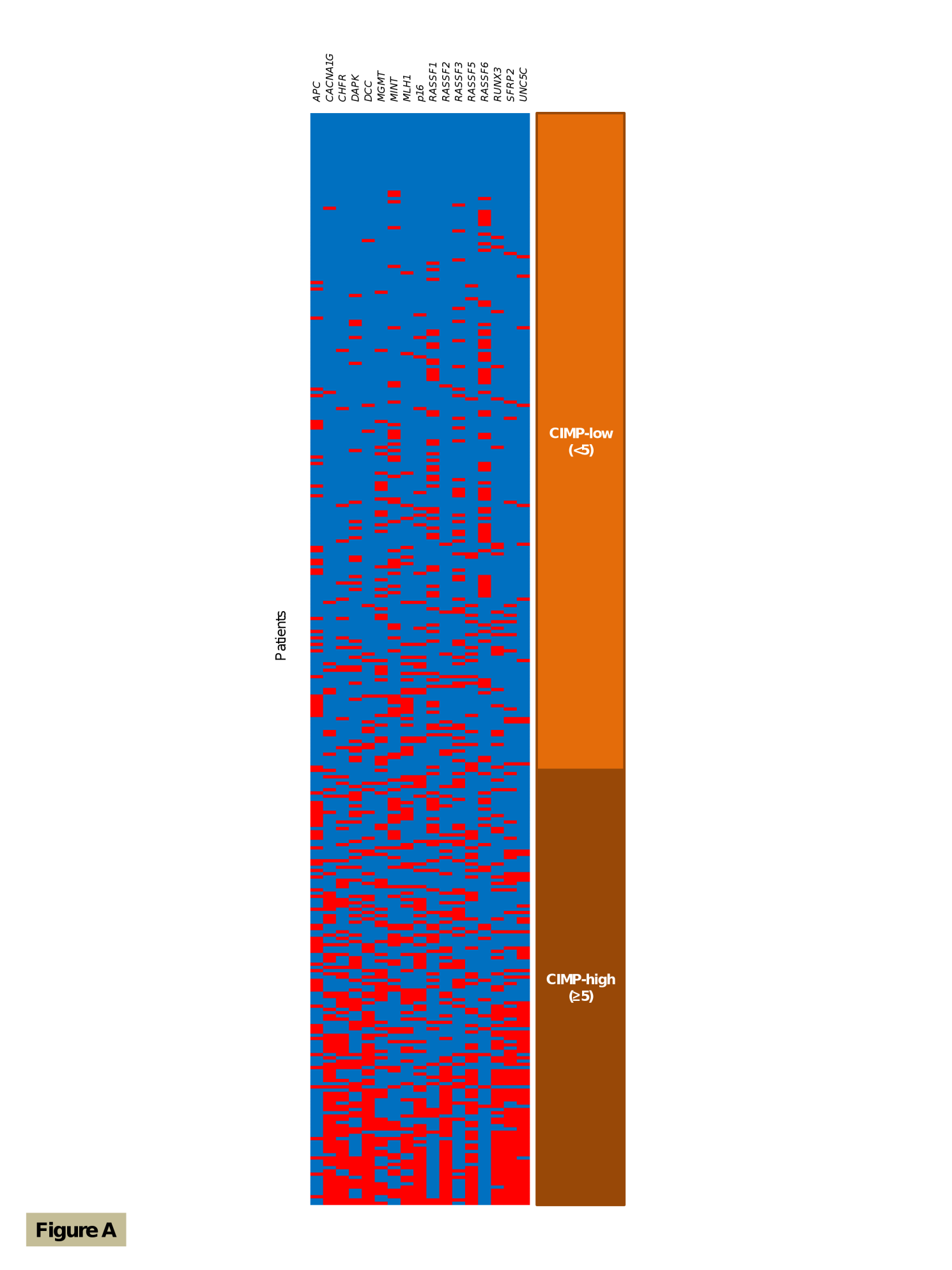


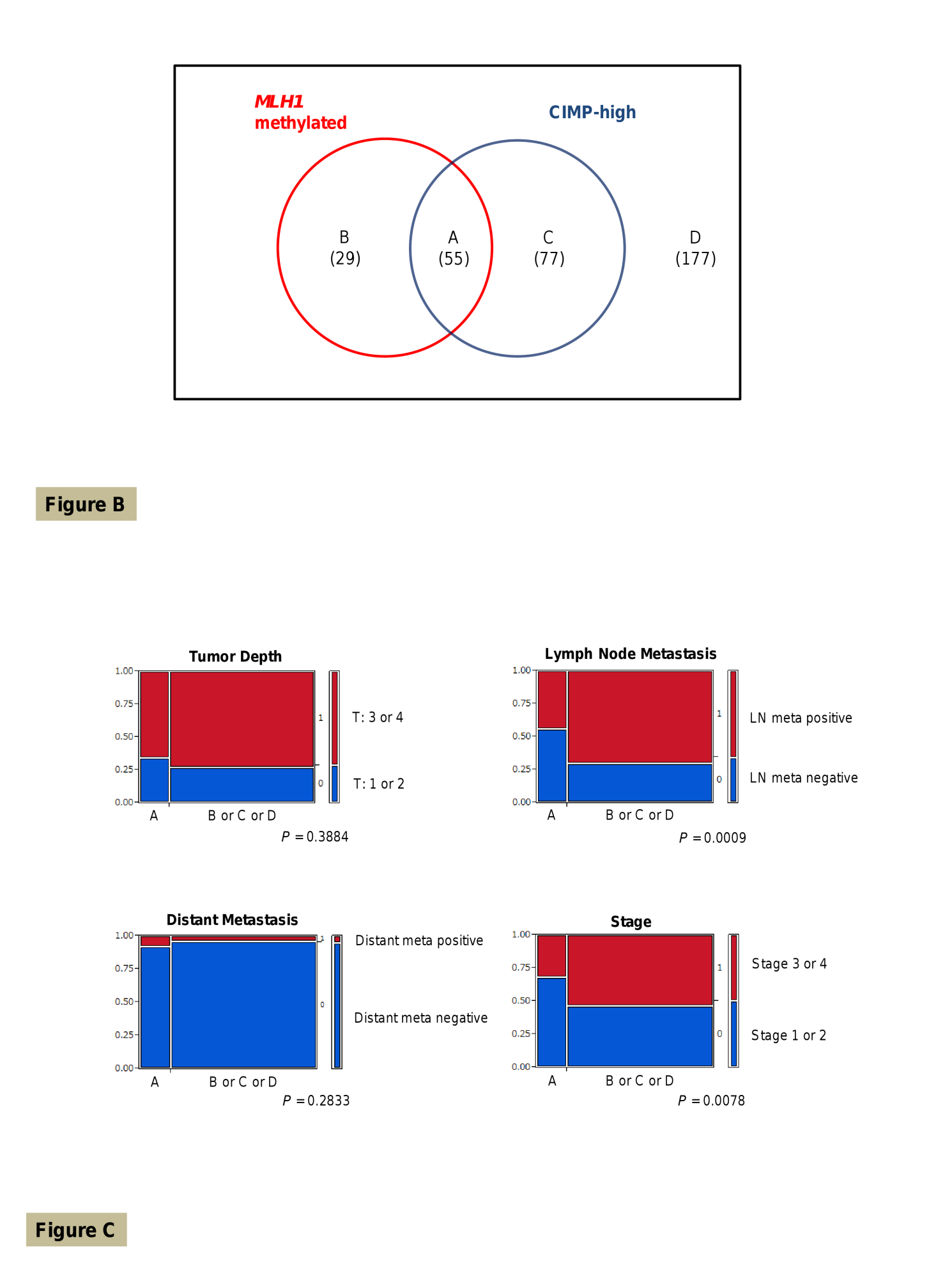

Supplement: S1 File — We investigated the methylation status of 17 promoter CpG island-related loci (APC, CACNA1G, CHFR, DAPK, DCC, MGMT, MINT, MLH1, p16, RASSF1, RASSF2, RASSF3, RASSF5, RASSF6, RUNX3, SFRP2, and UNC5C) in TCGA database (TCGA provisional). Upper 25% of each locus was determined as hyper-methylated. CIMP-high was defined as not less than 5 of the hyper-methylation of these loci (Figure A). The overlapping relationship between CIMP and MLH1 methylation status was analyzed. 55 patients were in the CIMP-high/MLH1 methylated, 29 patients were in the CIMP-low/MLH1 methylated, 77 patients were in the CIMP high/MLH1 non-methylated and 177 patients were in the CIMP-low/MLH1 non-methylated groups (Figure B). Correlation between tumor depth, lymph node metastasis, distant metastasis, Stage and CIMP/MLH1 methylation status were analyzed using Fisher’s exact test. Positive lymph node metastasis (p = 0.0009) and higher Stage (p = 0.0078) were positively correlated with CIMP-high/MLH1 methylated group (Figure C). (DOCX) [file pone.0130409.s001.docx]
