# Supplementary figures and images for: Clinical Significance of MLH1 Methylation and CpG Island Methylator Phenotype as Prognostic Markers in Patients with Gastric Cancer
Source: PLoS One. 2015 Jun 29;10(6):e0130409. doi: 10.1371/journal.pone.0130409 (PMC4488282; doi:10.1371/journal.pone.0130409)

**S2 File. Disease free survival in Gastric cancer patients in TCGA database**


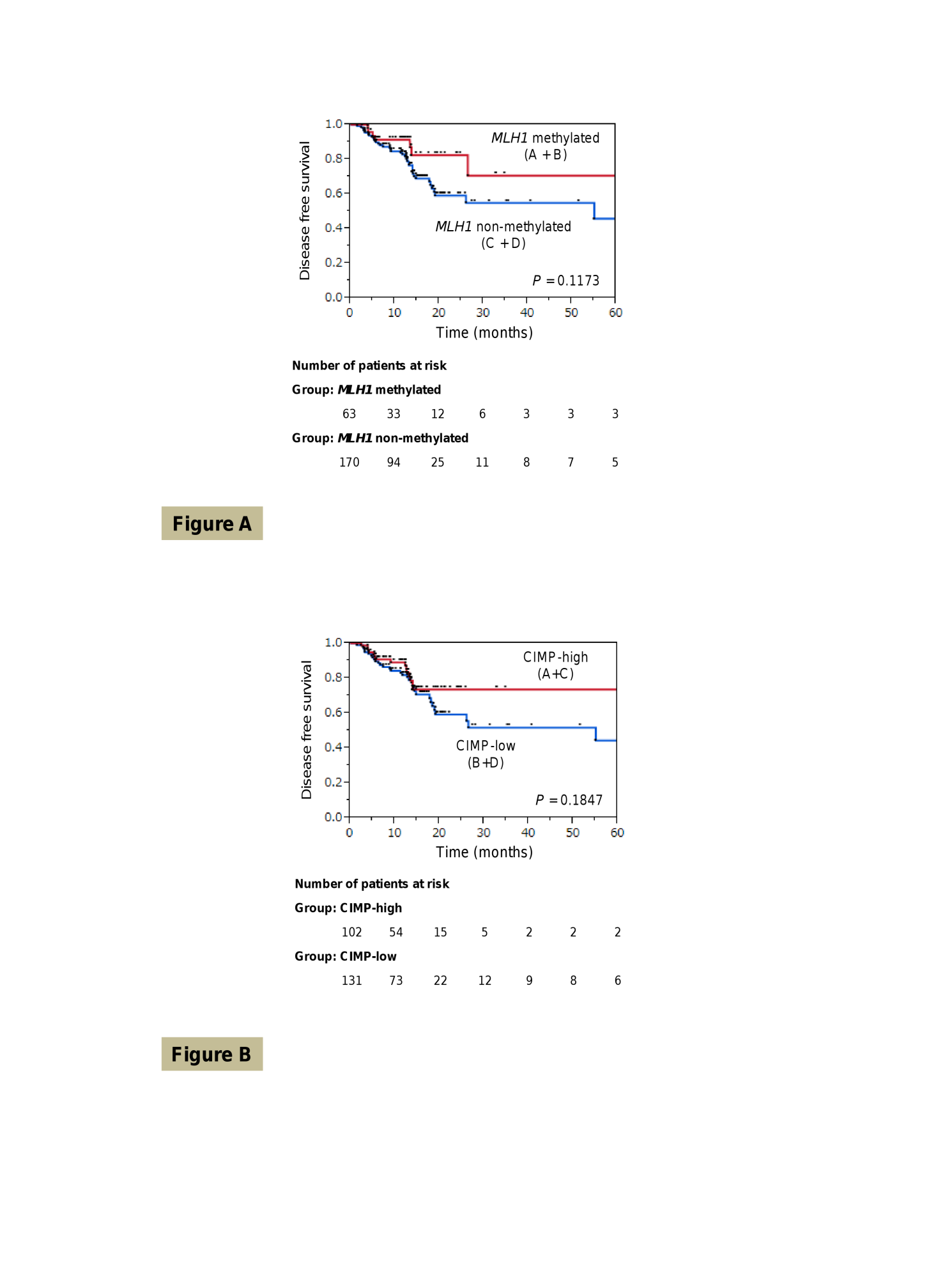


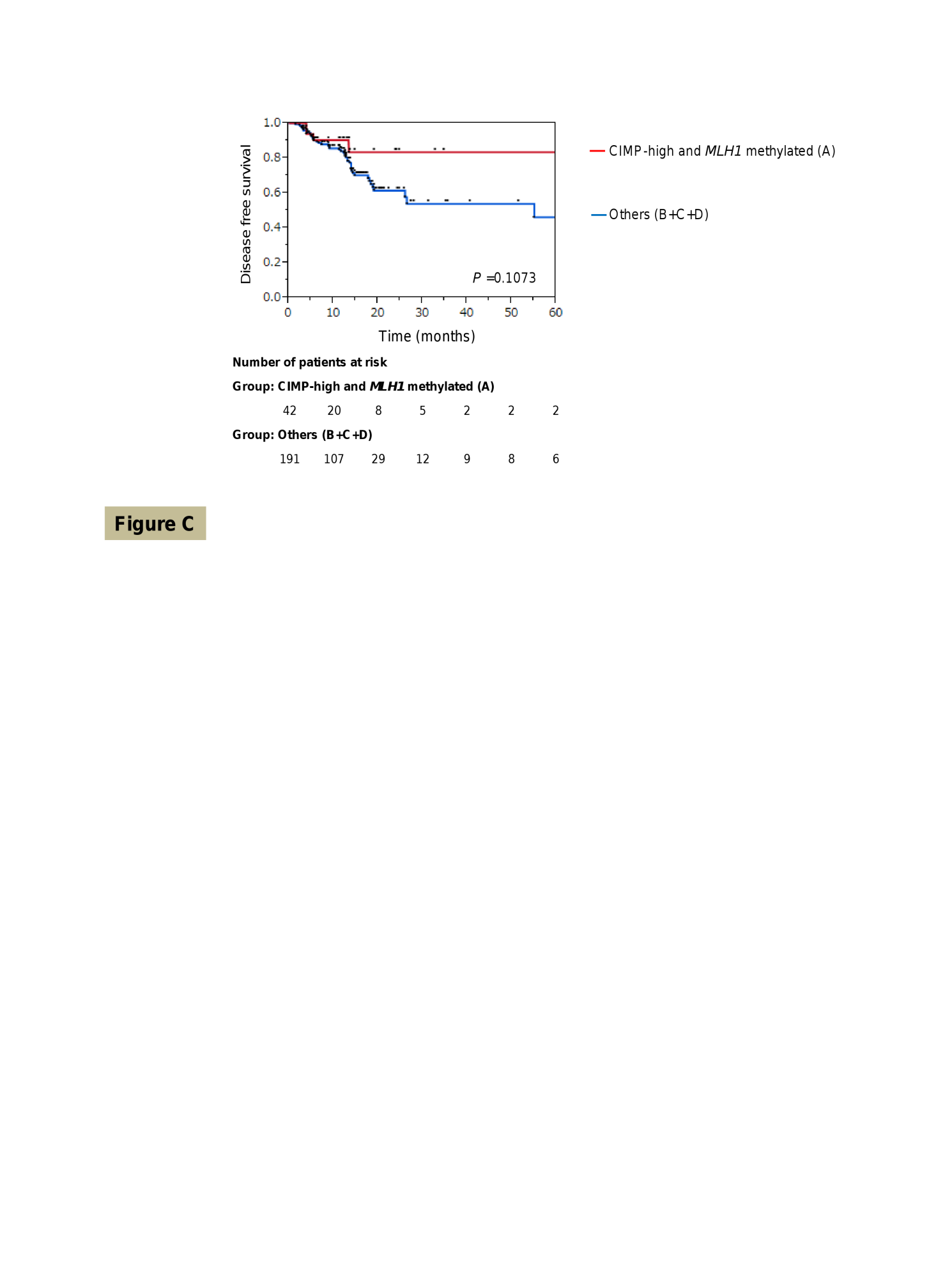

Supplement: S2 File — Kaplan—Meier survival curves were generated according to the MLH1 methylation status. The disease free survival rates were determined for the MLH1 methylated and non-methylated groups. Disease free survival rates were slightly higher in the MLH1 methylated group compared to the non-methylated group but the difference was not significant (log-rank P = 0.1173) (Figure A). Disease free survival rates were analyzed for the CIMP-high and CIMP-low groups, and the rate was slightly higher in the CIMP-high group than in the CIMP-low group but the difference was not significant (log-rank P = 0.1847) (Figure B). Disease free survival rates were analyzed and compared between CIMP-high/MLH1 methylated and other groups. We noted that the disease free survival rates were slightly higher in the combined CIMP-high/MLH1 methylated group, compared to the other groups where the differences were not statistically significant (log-rank P = 0.1073) (Figure C). (DOCX) [file pone.0130409.s002.docx]
