# Supplementary material for: Clinical Significance of MLH1 Methylation and CpG Island Methylator Phenotype as Prognostic Markers in Patients with Gastric Cancer
Source: PLoS One. 2015 Jun 29;10(6):e0130409. doi: 10.1371/journal.pone.0130409 (PMC4488282; doi:10.1371/journal.pone.0130409)
Supplement: S1 Table — (DOCX) [file pone.0130409.s003.docx]

**S1 Table. Primer sequences**

|  |  | **Gene** | **Forward primer sequence (5' - 3')** | | **Reverse primer sequence (5' - 3')** |  | **Restriction**  **enzyme** |  |
| --- | --- | --- | --- | --- | --- | --- | --- | --- |
|  | **Direct sequencing** | | |  |  |  |  |  |
|  |  | *KRAS* | | GCCTGCTGAAAATGACTGAA | AGAATGGTCCTGCACCAGTAA |  |  |  |
|  | **Bisulfite PCR** | | |  |  |  |  |  |
|  |  | *APC* | | GGTTTTGTGTTTTATTGYGGAGTG | CACCAATACAACCACATATCIATCAC |  | TaqI |  |
|  |  | *CACNA1G* | | GGGGGYGTTTTTTTTYGGATTTT | TTCCCCTACRCCCCTAAAACTTCC |  | EcoRI |  |
|  |  | *CHFR* | | AAA-ATCCTTAAAACTTCCAATCC | GTTAATTTTTTGT-YGTYGTTATTAATG |  | NruI |  |
|  |  | *COX2* | | GATTTGTAGTGAGYGTTAGGAGT | CCAAATACTCACCTATATAACTAAA |  | RsaI |  |
|  |  | *DAPK* | | TAGATTTTGTYGTTGYGAGTTGT | ATCCCCATTAACCRCCTACC |  | BstUI |  |
|  |  | *DCC* | | AAGGTGTTGGTTGAAATATGGAGAA | AAAAACCACTTACCRATTACTTAAAAA |  | HhaI |  |
|  |  | *HPP1* | | TGTTTAGTAGTTYGTTGTTYGGTTT | AACCCTCGCAAAATATCCAAC |  | NruI |  |
|  |  | *MGMT-Mp region* | | GAGGATGYGTAGATTGTTTTAG | AAACCRAAAACCTAAAAAAAAC |  | HhaI |  |
|  |  | *MGMT-Eh region* | | GTTTTTAGAAYGTTTTGYGTTT | CCTACAAAACCACTCRAAACTA |  | BstUI |  |
|  |  | *MINT1* | | GGGTTGGAGAGTAGGGGAGTT | CCATCTAAAATTACCTCRATAACTTA |  | TaqI |  |
|  |  | *MINT2* | | YGTTATGATTTTTTTGTTTAGTTAAT | TACACCAACTACCCAACTACCTC |  | TaqI |  |
|  |  | *MINT31* | | GAYGGYGTAGTAGTTATTTTGTT | CATCACCACCCCTCACTTTAC |  | HpyCH4IV |  |
|  |  | *MLH1 5*′ | | YGGGTAAGTYGTTTTGAYGTAGA | ATACCTAATCTATCRCCRCCTCATC |  | HhaI |  |
|  |  | *MLH1 3*′ | | GGGAGGGAYGAAGAGATTTAGT | ACCTTCAACCAATCACCTCAAT |  | RsaI |  |
|  |  | *p14* | | TTTYGGGGYGGAGATGGGT | ATCACCAAAAACCTACRCACCATATTC |  | TaqI |  |
|  |  | *p16* | | GGTTTTGGYGAGGGTTGTTT | ACCCTATCCCTCAAATCCTCTAAAA |  | TaqI |  |
|  |  | *RASSF1* | | TTAGTTTYGTTTTYGGGTTTTATAG | RCCCAACRAATACCAATCCC |  | HhaI |  |
|  |  | *RASSF2A-region1* | | TGAAGAGYGAGAGAAAAGAGAGGA | TCCAACCAAACTAAACAAACRATAA |  | HhaI |  |
|  |  | *RASSF2A-region2* | | TTGGGGAGGGTTTGATAGTTT | CRCACCCTACRCCCCTCTAAAA |  | HhaI |  |
|  |  | *RASSF3* | | GGGGYGGGGTTTAGGGAG | AACCCCRAACAATCCTTATACAACTAC |  | TaqI |  |
|  |  | *RASSF5* | | GTTTTYGTTTYGTTTAGTAGGTTG | AAAACRCCATAACCATACCC |  | TaqI |  |
|  |  | *RASSF6* | | AAAACRCCATAACCATACCC | CCATCRTTATTCRACTAAACTTAC |  | TaqI |  |
|  |  | *RUNX3* | | TTTTTYGTATTTTGTGAGTTGAGG | AAACACCAAAAACCAACCAACT |  | BstUI |  |
|  |  | *SFRP2-region1* | | GTYGGAGTTTTTYGGAGTTG | AACCCRCTCTCTTCRCTAAATAC |  | HhaI |  |
|  |  | *SFRP2-region2* | | GGTTGTTAGTTTTTYGGGGTTT | CAACIAACCAAAACCCTACAACAT |  | HhaI |  |
|  |  | *UNC5C* | | TTTAGTGGGGTTTTTAGTTGTTTG | TATCCCAATCCCAATCCRCAAC |  | HhaI |  |
|  |  | *3OST2* | | TTTGGTTAGTAGTTTTIGGAGAAGA | CCCTATAAACCATAACTCCATAAACC |  | TaqI |  |
|  |  | *FOXL2* | | TATAAAAAGTGATTTGGAGATGAATT | TCCCTAATACRCCAAAAACC |  | BstUI |  |
