# Supplementary material for: Clinical Significance of MLH1 Methylation and CpG Island Methylator Phenotype as Prognostic Markers in Patients with Gastric Cancer
Source: PLoS One. 2015 Jun 29;10(6):e0130409. doi: 10.1371/journal.pone.0130409 (PMC4488282; doi:10.1371/journal.pone.0130409)
Supplement: S2 Table — (DOCX) [file pone.0130409.s004.docx]

**S2 Table. Multivariate analysis of outcome predictors based on CIMP/MSI status**

|  |  |  |  |  | **Univariate** | | | |  | **Multivariate** | | |  |
| --- | --- | --- | --- | --- | --- | --- | --- | --- | --- | --- | --- | --- | --- |
|  | **Characteristic** |  | **Total** |  | **HR (95% CI)** |  | ***P*** |  |  | **HR (95% CI)** |  | ***P*** |  |
|  | **Age** |  |  |  |  |  | 0.4476 |  |  |  |  | 0.2941 |  |
|  | <70 |  | 45 |  | 1.00 (Referent) |  |  |  |  | 1.00 (Referent) |  |  |  |
|  | ≥70 |  | 23 |  | 1.30 (0.65–2.48) |  |  |  |  | 1.49 (0.70–3.12) |  |  |  |
|  |  |  |  |  |  |  |  |  |  |  |  |  |  |
|  | **Stage** |  |  |  |  |  | **0.0003** | *** |  |  |  | **0.0078** | ** |
|  | Stage I and II |  | 32 |  | 1.00 (Referent) |  |  |  |  | 1.00 (Referent) |  |  |  |
|  | Stage III and IV |  | 36 |  | 3.50 (1.75–7.49) |  |  |  |  | 2.75 (1.30–6.20) |  |  |  |
|  |  |  |  |  |  |  |  |  |  |  |  |  |  |
|  | **Differentiation** |  |  |  |  |  | **0.0032** | ** |  |  |  | **0.0101** | * |
|  | Well/Moderate |  | 32 |  | 1.00 (Referent) |  |  |  |  | 1.00 (Referent) |  |  |  |
|  | Poorly |  | 36 |  | 2.76 (1.39–5.83) |  |  |  |  | 2.51 (1.24–5.40) |  |  |  |
|  |  |  |  |  |  |  |  |  |  |  |  |  |  |
|  | ***KRAS* Mutation** |  |  |  |  |  | 0.1690 |  |  |  |  | 0.5526 |  |
|  | Absent |  | 65 |  | 1.00 (Referent) |  |  |  |  | 1.00 (Referent) |  |  |  |
|  | Present |  | 3 |  | 0.31 (0.02–1.47) |  |  |  |  | 0.56 (0.03–3.02) |  |  |  |
|  |  |  |  |  |  |  |  |  |  |  |  |  |  |
|  | **CIMP/MSI** |  |  |  |  |  | 0.0930 |  |  |  |  | 0.0562 |  |
|  | CIMP−/MSS |  | 36 |  | 1.00 (Referent) |  |  |  |  | 1.00 (Referent) |  |  |  |
|  | CIMP+/MSS |  | 22 |  | 0.74 (0.34–1.49) |  | 0.4027 |  |  | 0.61 (0.26–1.31) |  | 0.2068 |  |
|  | CIMP+/MSI |  | 8 |  | 0.24 (0.04–0.80) |  | **0.0173** | ***** |  | 0.21 (0.03–0.75) |  | **0.0123** | * |
|  | CIMP−/MSI |  | 2 |  | 1.55 (0.25–5.32) |  | 0.5810 |  |  | 1.71 (0.27–6.24) |  | 0.5078 |  |

* P < 0.05, ** P < 0.01, *** P < 0.001
